# Unraveling the Effects of Carotenoids Accumulation in Human Papillary Thyroid Carcinoma

**DOI:** 10.3390/antiox11081463

**Published:** 2022-07-27

**Authors:** Alessandra di Masi, Rosario Luigi Sessa, Ylenia Cerrato, Gianni Pastore, Barbara Guantario, Roberto Ambra, Michael Di Gioacchino, Armida Sodo, Martina Verri, Pierfilippo Crucitti, Filippo Longo, Anda Mihaela Naciu, Andrea Palermo, Chiara Taffon, Filippo Acconcia, Fabrizio Bianchi, Paolo Ascenzi, Maria Antonietta Ricci, Anna Crescenzi

**Affiliations:** 1 Department of Sciences, Roma Tre University, 00146 Rome, Italy; ros.sessa1@stud.uniroma3.it (R.L.S.); yle.cerrato@stud.uniroma3.it (Y.C.); michael.digioacchino@uniroma3.it (M.D.G.); armida.sodo@uniroma3.it (A.S.); filipo.acconcia@uniroma3.it (F.A.); paolo.ascenzi@uniroma3.it (P.A.); mariaantonietta.ricci@uniroma3.it (M.A.R.); 2 CREA (Council for Agricultural Research and Economics), Research Centre for Food and Nutrition, 00178 Rome, Italy; giovanni.pastore@crea.gov.it (G.P.); barbara.guantario@crea.gov.it (B.G.); roberto.ambra@crea.gov.it (R.A.); 3 Pathology Unit, Fondazione Policlinico Universitario Campus Bio-Medico, 00128 Rome, Italy; m.verri@policlinicocampus.it (M.V.); c.taffon@unicampus.it (C.T.); a.crescenzi@policlinicocampus.it (A.C.); 4 Unit of Thoracic Surgery, Fondazione Policlinico Universitario Campus Bio-Medico, 00128 Rome, Italy; p.crucitti@unicampus.it (P.C.); filippo.longo@policlinicocampus.it (F.L.); 5 Unit of Metabolic Bone and Thyroid Disorders, Fondazione Policlinico Universitario Campus Bio-Medico, 00128 Rome, Italy; a.naciu@policlinicocampus.it (A.M.N.); a.palermo@unicampus.it (A.P.); 6 Cancer Biomarkers Unit, Fondazione IRCCS Casa Sollievo della Sofferenza, 71013 San Giovanni Rotondo, FG, Italy; f.bianchi@operapadrepio.it

**Keywords:** antioxidant, carotenoid, papillary thyroid cancer, retinoic acid

## Abstract

Among the thyroid cancers, papillary thyroid cancer (PTC) accounts for 90% of the cases. In addition to the necessity to identify new targets for PTC treatment, early diagnosis and management are highly demanded. Previous data indicated that the multivariate statistical analysis of the Raman spectra allows the discrimination of healthy tissues from PTC ones; this is characterized by bands typical of carotenoids. Here, we dissected the molecular effects of carotenoid accumulation in PTC patients by analyzing whether they were required to provide increased retinoic acid (RA) synthesis and signaling and/or to sustain antioxidant functions. HPLC analysis revealed the lack of a significant difference in the overall content of carotenoids. For this reason, we wondered whether the carotenoid accumulation in PTC patients could be related to vitamin A derivative retinoic acid (RA) biosynthesis and, consequently, the RA-related pathway activation. The transcriptomic analysis performed using a dedicated PCR array revealed a significant downregulation of RA-related pathways in PTCs, suggesting that the carotenoid accumulation in PTC could be related to a lower metabolic conversion into RA compared to that of healthy tissues. In addition, the gene expression profile of 474 PTC cases previously published in the framework of the Cancer Genome Atlas (TGCA) project was examined by hierarchical clustering and heatmap analyses. This metanalysis study indicated that the RA-related pathways resulted in being significantly downregulated in PTCs and being associated with the follicular variant of PTC (FV-PTC). To assess whether the possible fate of the carotenoids accumulated in PTCs is associated with the oxidative stress response, the expression of enzymes involved in ROS scavenging was checked. An increased oxidative stress status and a reduced antioxidant defense response were observed in PTCs compared to matched healthy thyroids; this was possibly associated with the prooxidant effects of high levels of carotenoids. Finally, the DepMap datasets were used to profile the levels of 225 metabolites in 12 thyroid cancer cell lines. The results obtained suggested that the high carotenoid content in PTCs correlates with tryptophan metabolism. This pilot provided novel possible markers and possible therapeutic targets for PTC diagnosis and therapy. For the future, a larger study including a higher number of PTC patients will be necessary to further validate the molecular data reported here.

## 1. Introduction

In the past 30 years, the incidence of thyroid cancer (TC) has significantly increased, making it the fastest rising malignant solid tumor worldwide [1]. Among the TCs, papillary thyroid cancer (PTC) accounts for 90% of thyroid cancer cases [2]. In addition to the even more urgent necessity to discover new biological and therapeutic targets for PTC treatment, early diagnosis and management represent key points required for the improvement of patient prognosis. Indeed, the trauma caused by surgery and the recurrence of the tumor are still intractable issues, bringing a certain life and economic burden to patients [3]. Therefore, it is of great significance to further explore the pathogenesis of PTC and to find molecular targets with the potential for early diagnosis, prevention, and treatment. In recent years, Raman spectroscopy (RS) has emerged as a powerful tool for TC diagnosis. Indeed, our group has reported that the multivariate statistical analysis of RS spectra allows the discrimination of healthy from thyroid cancerous tissues and makes it possible to distinguish between the most common TC subtypes [4,5]. In particular, RS has allowed the identification of bands typical of carotenoids in PTC cells, suggesting that the presence of these molecules could represent a specific fingerprint of this type of TC and consequently a potential candidate for differential diagnosis [4,5,6].

Carotenoids are isoprenoids naturally occurring in fruits and vegetables and are synthesized in plants and microorganisms [7,8]. This wide family of C40 molecules has important functions not only in carotenoid-producing organisms, but also in animals that absorb these molecules in their diets. The primary carotenoids found in human plasma are α-carotene, β-carotene, γ-cryptoxanthin, lycopene, and lutein [9,10]. All these carotenoids display an antioxidant activity, and among them, α-carotene, β-carotene, and γ-cryptoxanthin are also precursors of vitamin A in animals [10]. Besides being essential for vision, the vitamin A derivative retinoic acid (RA) represents in vertebrates a major signal, controlling a wide range of biological processes thanks to its ability to bind two classes of nuclear receptors, i.e., the retinoic acid receptor (RAR) and the retinoid X receptor (RXR) [11,12,13]. RAR is part of the RAR/RXR heterodimer that binds DNA regulatory sequences, its RA-induced signaling regulating the transcription of genes involved in an array of biological processes such as pattern formation during embryonic development, cell differentiation, and control of certain metabolic activities [13].   

Here, we aim to determine in 11 PTC patients whether the carotenoid accumulated in this type of TC, as reported by RS analyses [4,5,6], is metabolized to RA or, rather, is used by cancer cells to support antioxidant functions. Considering the low availability of patients to be enrolled in this study, this was intended as a pilot study, providing a molecular fingerprint of PTC useful for the identification of novel possible markers for diagnosis and therapy.

## 2. Materials and Methods

### 2.1. Study Enrollment

We screened and enrolled subjects with thyroid nodular pathologies who were referred to the thyroid outpatient clinic of the Metabolic Bone and Thyroid Disorders Unit of Fondazione Policlinico Universitario Campus Bio-Medico (Rome, Italy) between January 2018 and January 2021. Our protocol adhered to the Declaration of Helsinki and to the International Conference on Harmonization Good Clinical Practice, receiving the approval of the local ethics committees. The participants were recruited from and were managed at Fondazione Policlinico Universitario Campus Bio-Medico (Rome, Italy), all of them granting written informed consent that allowed the use of their anonymized information for data analysis. The patient eligibility criteria were as follows: (i) ≥18 years old; (ii) one or more thyroid nodules with a medium-high ultrasound risk of malignancy (Thyroid Imaging—Reporting and Data System (TI-RADS) score ≥3) [14]; (iii) at least one thyroid nodule with a fine needle aspiration (FNA) report categorized as indeterminate, suspicious, or malignant and warranting thyroid surgery according to clinical guidelines [15]; (iv) final histological diagnosis of PTC; and (v) willingness to provide material for RS and carotenoid analysis. Once thyroid histology was reported and RS analysis performed, only those subjects with material available for the transcriptomic and proteomic analyses were included in the final analysis (Table 1). 

### 2.2. Patients’ Evaluation

All subjects were submitted to thyroid US evaluation, using a frequency range of 10–12 MHz on a MyLab 50 (Esaote, Genova, Italy). The US scans of the thyroid gland and neck area were performed by 2 experienced endocrinologists at the Endocrinology Unit at Fondazione Policlinico Universitario Campus Bio-Medico (Rome, Italy). Nodules were classified according to the American College of Radiology (ACR) TI-RADS risk stratification criteria [14] without prior knowledge of the cytological results. Patients harboring one or more thyroid nodules of medium-high malignancy risk by US (TI-RADS score ≥ 3) and submitted to thyroid FNA represented the criteria for patient selection. Once thyroid FNA cytology was reported, only those subjects with at least one nodule categorized as indeterminate, suspicious, or malignant, with a formal indication of thyroid surgery according to the international guidelines [15], and a final histological diagnosis of PTC were enrolled in the study. Histological diagnosis was reported in agreement with the current edition of the World Health Organization (WHO) classification of endocrine tumors [16]. Because of the small size of the available tumor tissues explanted from patients, it was not always possible to perform all the transcriptomic and proteomic analyses on the same samples. 

### 2.3. Raman Spectroscopy 

Raman spectra were collected using a Renishaw InVia Micro-Raman spectrometer, equipped with a solid-state diode laser source at 532 nm (nominal output power of 100 mW) and a confocal microscope (Leica 50× Long Working Distance objective), used both to focalize the incident laser beam and to collect the back-scattered light. Elastically scattered light is rejected by using a holographic edge filter, while inelastically scattered intensity is dispersed by a diffraction grating (1800 grooves/mm) on a Peltier cooled 1024 × 256 pixel Charge-Coupled Device (CCD) detector. The final instrumental resolution is of the order of 1 cm^−1^. To prevent photo damage, the laser power at the sample was controlled by neutral density filters. The spectra were collected in the 100–3600 cm^−1^ range. On each sample, 5 measurement points were selected and 5 scans per point were accumulated for a total integration time of 50 s. The experimental conditions and data acquisition were controlled by Wire (Renishaw) software, which also allowed the preliminary data reduction (e.g., background and fluorescence subtraction). The MATLAB and ORIGIN 9.0 software were used to analyze the data.

### 2.4. Extraction of Carotenoids from the Thyroid Tissue

The carotenoids extraction was performed according to the procedure proposed by Peng and colleagues [17]. Briefly, healthy and tumoral thyroids were weighted and incubated in 560 μL of phosphate buffered saline solution containing 1 mg of butylated hydroxytoluene (BHT, Merck KGaA, Darmstadt, Germany) and 70 μL of a collagenase (50 mg/mL; Carlo Erba, Milano, Italy) at 37 °C for 1 h using a shaker (500 rpm). After a manual homogenization using glass potters, 70 μL proteinase K (20 mg/mL; Merck KGaA) was added to the samples, which were further incubated at 37 °C for 30 min using a shaker. After vortexing for 1 min, the lysate was mixed with 700 μL of an ethanolic buffer containing 1% SDS and 0.1% BHT. The solution was vortexed with n-hexane (Merck KGaA) and then centrifugated at 13,000 rpm for 30 s. The extraction with n-hexane was repeated twice, and the supernatants were then collected and dried under a nitrogen atmosphere. The extracts were solubilized in 120 μL methanol:chloroform 1:1, 80 μL of which were immediately injected into the High Performance Liquid Chromatography (HPLC). All the procedures were performed in the dark, under red light. 

### 2.5. High Performance Liquid Chromatography (HPLC)

Carotenoids were separated following the method from [18] with minor modifications, using a YMC C30 column (250 mm × 4.6 mm i.d., 5 µm particle size) (YMC, Kyoto, Japan) kept at 35 °C and a flow of 1 mL/min. The mobile phase consisted of: (A) methanol, and (B) tert-butyl methyl ether. The following gradient was used (35 min): 90% A for 2 min, followed by a linear gradient to 80% A for 8 min and then to 30% A for 10 min, which was then maintained for further 15 min. The photodiode array was set at 450 and 472 nm. α-carotene, β-carotene, lutein, and lycopene were recognized and quantified by comparison with analytical standards (Merck KGaA). Unidentified peaks were quantified using the calibration curve of β-carotene. To verify the quantification reliability of using a few tens of mg of tissue, the method was verified by measuring the recovery of carotenoids from tissues spiked with known concentrations of β-carotene (the recovery rate was 96 ± 10%) and by performing up to four serial repetitions of n-hexane extraction (the extractions beyond the second did not yield detectable β-carotene).

### 2.6. Total RNA Extraction

Total RNA was extracted from the healthy and pathological lobes of each patient. RNA was obtained from two formalin-fixed paraffin-embedded (FFPE) tissue sections of 5 µm thickness each, using the RNAeasy^®^ FFPE kit (Qiagen, Hilden, Germany) and following the manufacturer’s instructions. Briefly, sections were deparaffinized using heptane and methanol. After centrifugation, pellets were air-dried and then resuspended into PKD buffer (Qiagen) containing 10 µL proteinase K (>600 mAU/mL; provided with the kit). After a first incubation at 56 °C for 15 min and a second incubation at 80 °C for 15 min, samples were put on ice for 3 min and finally centrifuged at 13,000 rpm for 15 min. The DNA was removed by incubation of the collected supernatant with DNAse for 15 min. After the addition of RBC buffer, samples were loaded on a RNeasy MinElute spin column and centrifuged. Finally, RNA was eluted using 30 µL RNase-free water, and quantified with Nanodrop 2000 (Thermo Fisher Scientific, Waltham, MA, USA).

### 2.7. Transcriptomic Analysis Using the PCR Array “Human Retinoic Acid Pathway”

Three micrograms of total RNA were reverse transcribed using the RT2 First Strand Kit (Qiagen), following the manufacturer’s instructions. The expression of 96 genes was analyzed using the RT2 Profiler “Human Retinoic Acid Pathway” PCR Array 96-well format (cat no. 330231 PAHS-180Z; Qiagen) in combination with the RT2 SYBR Green PCR Master Mix (Qiagen), according to the manufacturer’s protocol. Of these 96 genes, 84 genes belong to the human RA-related pathways, and 12 genes are either housekeeping genes (i.e., ß-actin (ACTB), ß-2-microglobulin (B2M), glyceraldehyde-3-phosphate dehydrogenase (GAPDH), hypoxanthine phosphoribosyltransferase 1 (HPRT1), and ribosomal protein lateral stalk subunit P0 (RPLP0)) or internal controls (i.e., human genomic DNA contamination, reverse transcription control, positive PCR control). The AriaMx Real-time PCR system (Applied Biosystems, Waltham, MA, USA) was used and the three steps of the cycling program were: 95 °C for 10 min for 1 cycle, followed by 40 cycles of 95 °C for 15 s and 60 °C for 1 min. The expression levels of each gene were quantified and reported as relative quantity (RQ) with respect to the calibrators represented by the housekeeping genes, according to the 2^−ΔΔCt^ method. Data analyses were performed using the Agilent AriaMx 1.71 software (Thermo Fisher Scientific) and the GeneGlobe software (Qiagen). The analysis of the differentially expressed genes (DEGs) between the healthy and PTC thyroids was performed using Venn diagrams (Bioinformatics & Evolutionary Genomics, http://bioinformatics.psb.ugent.be/webtools/Venn/; accessed on 13 January 2022). Visualization, interpretation, and analysis of the biomolecular pathways were performed using Reactome (https://reactome.org; accessed on 13 January 2022), a free, open-source, open-data, curated, and peer-reviewed knowledgebase [19].

### 2.8. Quantitative Real-Time Reverse Transcription–Polymerase Chain Reaction (qRT-PCR)

Total RNA was reverse transcribed and amplified with a GoTaq 2-step RT-qPCR system (Promega, Madison, WI, USA), following the manufacturer’s instructions. The cDNA obtained was then amplified for the following transcripts: CRABP2 (forward: 5′-AGTGTCCAGTGCTCCAGCCTA-3′; reverse: 5′-CTGCAGCCACAGCAATCTTC-3′), CYP26B1 (forward: 5′-AGCTAGTGAGCACCGAGTGG-3′; reverse: 5′-GGGCAGGTAGCTCTCAAGTG-3′), DHRS3 (forward: 5′-CGTTGCTGGCAATCAGATCG-3′; reverse: 5′-CGCGGTTTCAAAGTGCAAGA-3′), RARγ (forward: 5′-CAGAGCAGCAGTTCTGAAGAGATA-3′; reverse: 5′-GACACGTGTACACCATGTTCTTCT-3′), RDH10 (forward: 5′-TGGGACATCAACACGCAAAGC-3′; reverse: 5′-TGCAAGTTACAGTGGGGCAGA-3′), RET (forward: 5′-CGCGACCTGCGCAAA-3′; reverse: 5′-CAAGTTCTTCCGAGGGAATTCC-3′), RXRβ (forward: 5′-AGCTCCCCCAGGATTCTC-3′; reverse: 5′-CAGGGAGTGACACTGTTGAGTTA-3′). The amplifications were performed using the SYBR Green system and the Rotor Gene RG-6000 Real-Time Thermocycler (Corbett Research, Qiagen GmbH), using the following thermal cycling conditions: 95 °C for 2 min followed by 40 cycles at 95 °C for 15 s and 60 °C for 1 min. The data were reported as RQ with respect to a calibrator sample (i.e., ACTB), according to the 2^−ΔΔCt^ method. 

### 2.9. Biochemical Analysis

The frozen thyroids were weighted, homogenized, and sonicated in lysis buffer (20 mM Tris-HCl pH 8.0, 137 mM NaCl, 10 mM EDTA, 10% glycerol (*v*/*v*), 1% Triton™ X-100 (*v*/*v*), and protease inhibitors). Fifteen micrograms of protein extracts, previously quantified with the Bradford assay (Bio-Rad Laboratories, Hercules, CA, USA), were resolved by SDS-PAGE and transferred onto polyvinylidene fluoride (PVDF) membranes (Bio-Rad Laboratories). As reported elsewhere [20], after blocking with 3% BSA (*w*/*v*) dissolved in TBS buffer/0.5% Tween-20 (*v*/*v*), the membranes were probed overnight at 4 °C with the following antibodies purchased from Santa Cruz Biotechnology (Dallas, TX, USA): anti-catalase (CAT; sc-365738), anti-glutathione peroxidase-4 (GPx-4; sc-166570), anti-heme oxygenase-1 (HO-1; sc-136960), anti-NADPH oxidase 4 (NOX4; sc-518092), anti-NAD(P)H quinone dehydrogenase 1(NQO1; sc-32793), anti-oxoguanine glycosylase 1 (OGG1; sc-376935), anti-superoxide dismutase-1 (SOD-1; sc-271014), anti-vinculin (sc-73614), anti-ß-actin (sc-47778), and anti-γH2AX (sc-517348). The membranes were then incubated for 1 h at room temperature with an anti-mouse HRP-conjugated secondary antibody (Bio-Rad Laboratories). All the experiments were technically repeated at least twice. Blots were acquired and processed using the ChemiDoc™ Imaging system (Bio-Rad Laboratories) [21]. Protein quantification was performed using the Image Lab software (version 6.0.1, Bio-Rad Laboratories). 

### 2.10. Immunohistochemistry Analysis

At the time of surgery, the removed specimens were immediately submitted unfixed to the Pathology Unit at Fondazione Policlinico Universitario Campus Bio-Medico (Rome, Italy) in an appropriately labelled container. After gross evaluation, the specimens were formalin fixed, sampled, and processed for paraffin embedding. Four-micron sections from the paraffin block of the formalin-fixed specimens were used for immunohistochemical stain. Immunohistochemistry was performed with an automatized instrument (Dako Omnis, Agilent, Santa Clara, CA, USA) using an anti-SR-B1 rabbit monoclonal (clone EP1556Y, Abcam, Cambridge, UK) and revealed with peroxidase development. Negative controls were obtained by omitting the primary antibody; the positive control consisted of liver tissue. The thyroid lesions were subjected for SR-B1 immunohistochemistry. For each lesion, the section comprised both pathological tissue and normal parenchyma. Immunohistochemical evaluation was conduct in a blind way by two pathologists. Images were acquired using a Nikon Eclipse Ni microscope equipped with a Nikon Digital Camera DS-Fi3. The original magnification was 20× (Nikon, Tokyo, Japan). 

### 2.11. Gene Expression Analysis of External Database Repository

Gene expression normalized data (RNAseq) of the 474 PTC cases of the Cancer Genome Atlas (TGCA) project were downloaded from cBioPortal (https://www.cbioportal.org/datasets; accessed on 26 January 2022) [22]. The data were trimmed +1 to eliminate 0 values before statistical analyses. The gene expression data were median centered and log2 transformed. Hierarchical clustering and heatmap analyses were performed using Cluster 3.0 for Mac OS X (C Clustering Library 1.56; http://bonsai.hgc.jp/~mdehoon/software/cluster/software.htm; accessed on 26 January 2022) and Java Tree View (Version 1.1.6r4; http://jtreeview.sourceforge.net; accessed on 26 January 2022). The uncentered correlation and centroid linkage clustering method was used. Statistical analyses (including contingency plot) were performed using JMP 16 (SAS Institute S.R.L., Milano, Italy).

### 2.12. Statistical Analysis

The data were analyzed using GraphPad Prism 6 (version 6.01, GraphPad Software Inc., San Diego, CA, USA). The data were expressed as mean values ±standard deviations (SD). The statistical significance of differences was tested using the Student’s two-tailed *t*-test (df: degree factor). *p* values of less than 0.05 were considered statistically significant. 

## 3. Results

As previously reported, the Raman spectra of PTC tissues differ from those collected from both healthy thyrocytes and cells affected by other types of cancer [4]. In Supplementary Figure S1 is reported an exemplificative Raman spectrum derived from the TIR48 PTC patient. In the fingerprint region, the Raman spectra of PTC tissues are dominated, compared to the healthy counterpart, by intense bands at 1003, 1155, and 1515 cm^−1^. These bands are due to the resonance effect of free carotenoids when excited at 532 nm. Other bands at 2155, 2301, and 2652 cm^−1^, also related to carotenoids, are visible, along with bands at 1444, 1655, and 2848 cm^−1^. The latter three bands are ascribed to fatty acid vibrational modes, namely CH_2_ wagging, CC asymmetric stretching, and CH_2_ symmetric stretching, respectively. With the aim of dissecting the molecular effects of carotenoid accumulation in PTC patients, here we performed a combination of analyses (i.e., HPLC, transcriptomic, oxidative stress analysis, and metanalysis) to clarify whether carotenoids are required to provide increased retinoic acid (RA) synthesis and signaling and/or to sustain antioxidant functions.

### 3.1. Determination of Carotenoids Content in Healthy and PTC Thyroids by HPLC

**Figure 1:**
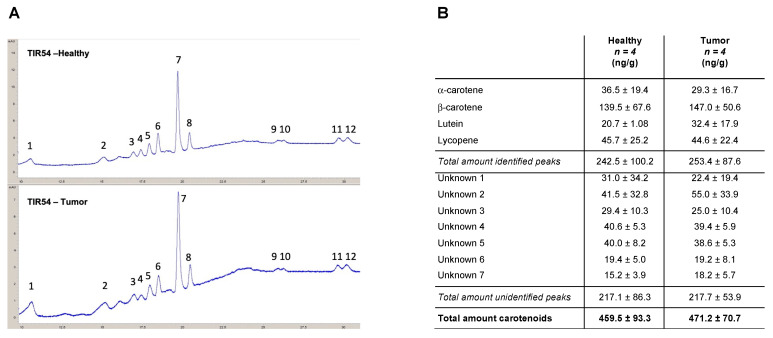
HPLC profiles of carotenoids recorded in the healthy and tumoral thyroid lobes of PTC patients. (**A**) Representative carotenoids HPLC chromatogram: (1) lutein; (2) unknown 1; (3) unknown 2; (4) unknown 3; (5) unknown 4; (6) α-carotene; (7) β-carotene; (8) unknown 5; (9) unknown 6; (10) unknown 7; (11 and 12) lycopene. (**B**) Mean values of α-carotene, β-carotene, lutein, and lycopene concentrations (ng/g of thyroid tissue) quantified in the healthy and pathological lobes of the thyroids derived from four PTC patients (i.e., TIR22, TIR54, TIR77, and TIR94), together with seven unidentified peaks attributable to molecules with absorbances measured at 450 and 472 nm.

The presence of carotenoids in PTC patients was corroborated using the HPLC analyses of thyroids (Figure 1A). α-carotene, β-carotene, lutein, and lycopene were detected in the thyroids of all the four patients analyzed, together with five unidentified peaks attributable to molecules with absorbances measured at 450 and 472 nm (Figure 1B and Supplementary Table S1). The obtained results indicated that β-carotene (139.5 ± 67.6 and 147.0 ± 50.6 ng/g in healthy lobes and PTCs, respectively) is the most prevalent carotenoid found in healthy and PTCs thyroid lobes of the four analyzed patients (Figure 1B). Overall, for all the identified carotenoids, no significant differences were found between healthy thyroid lobes and the PTC counterparts of the analyzed patients (Figure 1B). Notably, no significant differences were scored between the total amount of the identified carotenoids (i.e., α-carotene, β-carotene, lutein, and lycopene) (242.5 ± 100.2 and 253.4 ± 87.6 ng/g in healthy lobes and PTCs, respectively) and the unidentified carotenoids (i.e., unknown 1 to 7) (217.1 ± 86.3 and 217.7 ± 53.9 ng/g in healthy lobes and PTCs, respectively) (Figure 1B and Supplementary Table S1).

Of note, the carotenoids are Raman resonant only as free molecules and not when they are bound to other biomolecules, and HPLC measures the total amount of carotenoids irrespective of their binding status. Therefore, the discrepancy between the Raman and the HPLC analyses could be ascribed to the free/bound state of carotenoids in healthy versus PTC samples.

### 3.2. Evaluation of Carotenoids Metabolism in Healthy and PTC Thyroids

To further investigate the reason why the Raman spectra showed a marked increase in the band corresponding to free carotenoids and considering that β-carotene was the prevalent compound among the carotenoids identified by HPLC in the four analyzed patients, we tested the possibility that the different distributions of carotenoids between the healthy and the PTC counterparts could be due to a different metabolism of provitamin A carotenoids and more in the detail of the RA biosynthesis and related metabolism.

**Figure 2:**
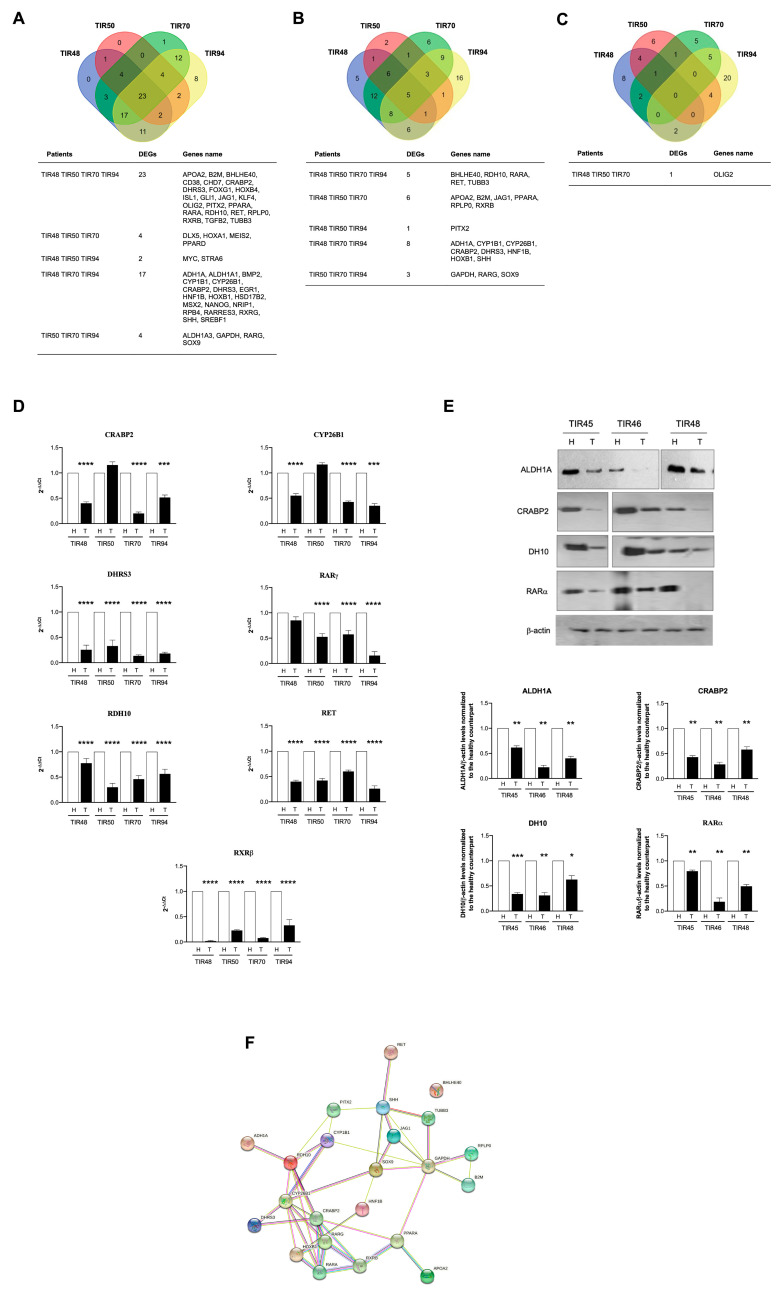
Transcriptome data analysis derived from PTC patients. Four PTC patients (i.e., TIR48, TIR50, TIR70, and TIR94) were analyzed by comparing the healthy and pathological lobes. (**A**) Venn diagram of the differentially expressed genes (DEGs) that were found significantly modulated in the four PTC patients analyzed. Twenty-three DEGs were found significantly modulated in all four of the patients, and a further 27 DEGs were found modulated in at least three patients (foldchange (FC)| >1.5|). (**B**) Venn diagram of the significantly down-regulated DEGs. Among the overall 23 transcripts significantly downregulated, five were found downregulated in all the three PTC analyzed patients and 18 were downregulated in at least three out of four PTC patients. (**C**) Venn diagram of the only significantly up-regulated DEG. Venn diagrams were calculated and drawn using the software available at the Bioinformatics & Evolutionary Genomics website (http://bioinformatics.psb.ugent.be/webtools/Venn/; accessed on 13 January 2022). (**D**) To validate DEGs, RT-qPCR experiments were performed using the RNA extracted from the healthy and PTC lobes of TIR48, TIR50, TIR70, and TIR94 patients. The expression levels of CRABP2, CYP26B1, DHRS3, RARγ, RDH10, RET, and RXRß genes have been reported as relative quantity in the PTC lobe with respect to the healthy one for each patient analyzed, according to the 2^−ΔΔCt^ method. Data are reported as mean ±SD of experiments repeated at least three times (Student’s *t*-test, **** *p* < 0.0001, *** *p* < 0.001, with respect to the relative healthy lobe). (**E**) To validate DEGs, immunoblot experiments were performed using the protein lysates derived from the healthy and PTC lobes of TIR45, TIR46, and TIR48 patients. The expression levels of ALDH1A, CRABP2, DH10, and RARα proteins in the PTCs lobes have been normalized to the healthy lobe of each patient analyzed. Both representative images of the immunoblot and their quantification are reported. Graphs illustrate the mean ± SD of experiments repeated at least three times (Student’s two-tailed *t*-test, * *p* < 0.05; ** *p* < 0.01; *** *p* < 0.001, with respect to the relative healthy lobe). (**F**) Protein–protein interactions network involving the DEGs significantly modulated in the PTC patients were identified using the STRING database. A PPI network with 22 interaction pairs of the DEGs was identified.

The gene expression profile related to the human RA pathway was performed in matched healthy and PTC counterparts by the RT^2^ Profiler PCR Array “Human Retinoid Acid Pathway”. The Supplementary Table S2 shows the complete list of the genes significantly modulated (fold change (FC)| >1.5|) in each analyzed patient (i.e., TIR48, TIR50, TIR70, and TIR94). Supplementary Figures S2 and S3 show the heatmaps and the scatter plot, respectively, of the gene expression profile in each patient. Overall, compared to the control, 23 differentially expressed genes (DEGs) were found significantly modulated in all four patients and further 27 DEGs were found modulated in at least three patients (FC| >1.5|) (Figure 2A). Of these, five genes were downregulated in all the four analyzed patients and 18 were downregulated in at least three out of the four patients (Figure 2B). Only one gene was commonly upregulated in three out of four PTC patients (Figure 2C). 

To validate the RT^2^ Profiler PCR Array results, RT-qPCR and immunoblot experiments were performed on selected DEGs. CRABP2, CYP26B1, DHRS3, RARγ, RDH10, RET, and RXRß were validated by RT-qPCR in the same patients tested by the RT^2^ Profiler PCR Arrays (Figure 2D). In addition, the ALDH1A, CRABP2, DH10, and RARα protein levels were checked by immunoblots performed using the healthy and pathological lobes of the thyroids of three further PTC patients (i.e., TIR45, TIR46, TIR48) (Figure 2E). The results obtained confirmed the downregulation of selected DEGs in the PTC lobes compared to the matched healthy ones.

To perform an unsupervised discovery analysis of the pathways significantly modulated because of gene expression modulation in the analyzed patients, we used the Reactome software. The obtained results showed that both the overall significantly modulated DEGs as well as the significantly downregulated DEGs were enriched in several biological pathways, as reported in Supplementary Tables S3 and S4. In particular, “Signaling by RA”, “RA biosynthesis pathway”, “Signaling by nuclear receptors”, “BMAL1:CLOCK, NPAS2 activates circadian gene expression”, and two pathways related to HOX genes expression (“Activation of anterior HOX genes in hindbrain development during early embryogenesis” and “Activation of HOX genes during differentiation”) were significantly downregulated and displayed low false discovery rate (FDR) values in PTCs compared to their relative counterparts (Supplementary Table S4). Protein–protein interaction networks involving the DEGs significantly modulated in the PTC patients were identified using the STRING database ([23]). A PPI network with 22 interaction pairs of the DEGs was identified (Figure 2F). 

Mammalian scavenger receptors class B type 1 (SR-B1) expression is essential for the cellular uptake of carotenoids [24,25]. The immunohistochemistry analysis of the SR-B1 receptor showed that all the examined lesions, as well as the normal parenchyma, did not show any SR-B1 expression in thyrocytes (Supplementary Figure S4). This suggests that the increase in carotenoids levels as detected by RS [4,5] was not due to an increased uptake but possibly by a different carotenoid metabolism between the healthy and PTC lobes. 

Overall, the obtained results indicate that despite HPLC analysis showed a comparable content of carotenoids in heathy and matched PTC thyroid lobes, the precursors of vitamin A in animals (i.e., α-carotene, β-carotene, and γ-cryptoxanthin) were converted to RA to a lower extent in PTC compared to healthy matched lobes [10]. This may explain the higher levels of free carotenoids detected by RS in PTC compared to the healthy counterparts [4,5], also considering that we did not observe an increased expression of the carotenoids receptor SR-B1.

### 3.3. Impact of Retinoid Acid-Signature in a Cohort of Patients with Papillary Thyroid Cancer 

**Figure 3:**
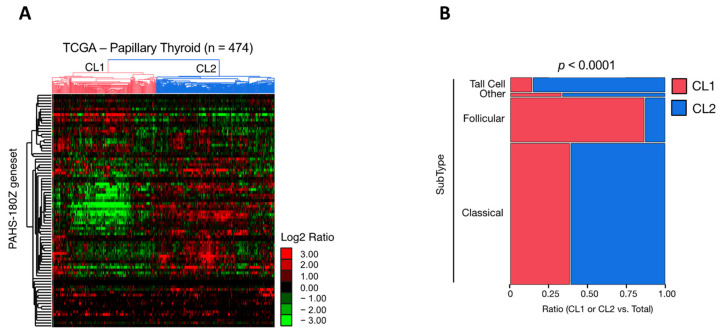
Gene expression metanalysis of external datasets using the RA-regulated genes. (**A**) Hierarchical clustering of “Human Retinoid Acid Pathway”-regulated genes in the framework of the TCGA-PTC dataset representing 474 PTC cases [22]. (**B**) Contingency analysis of PTC subtype distribution in CL1 or CL2 samples as in (**A**). Significance was calculated by the Likelihood Ratio test (JMP 16.0; SAS).

Because the study may suffer due to the low number of available patients, to further investigate the biological and pathological significance of our identified “Human Retinoid Acid Pathway” signature we evaluated the expression of 84 RA-related genes derived from the 96-well PCR array in the framework of the Cancer Genome Atlas (TGCA) project [22] (Supplementary Table S5). An unsupervised hierarchical clustering analysis was performed to investigate the human RA pathway gene signature (n = 84) in the TCGA-PTC dataset (Figure 3). The obtained results revealed that within the 474 PTC available cases, the 84 gene RA pathway signature allowed the identification of two main clusters (i.e., Cluster 1 and 2) characterized by a heterogenous gene expression pattern (Figure 3A). Cluster 1 includes 225 PTC cases, whereas Cluster 2 includes 248 PTC cases. Besides PTC, the other two categories of TC with a larger incidence are the follicular variant of PTC (FV-PTC) and the follicular thyroid carcinoma (FTC). Intriguingly, FV-PTC (n = 99) failed predominantly in Cluster 1 (n = 86) compared to Cluster 2 (n = 13) (Figure 3B). Conversely, Cluster 2 was characterized mainly by the classical (n = 190) and tall cell (n = 29) PTC subtypes (Figure 3B). The analysis of the gene expression FC between Cluster 1 and Cluster 2 revealed that among the 84 genes belonging to the “Human Retinoid Acid Pathway”, 58 were significantly modulated (*p* < 0.05). Among these, 42 genes (72.4%) were significantly downregulated in Cluster 1 compared to Cluster 2 (*p* < 0.05, Welch’s *t*-test) (Supplementary Table S6). 

Overall, these analyses allow the speculation that the subset of PTCs in which the RA-related pathways result in being significantly downregulated correlates more with FV-PTC, which was reported to have an intermediate clinical behavior between classical PTC (C-PTC) and FTC [26]. 

### 3.4. Antioxidant Response in Healthy and PTC Thyroids

Carotenoids are known to be potent scavengers of reactive oxygen species (ROS), thus providing protection against oxidative damage to photosynthetic and non-photosynthetic organisms at all levels of complexity [9,10,27,28,29,30]. However, newly formed carotenoids radical products can undergo further transformations, leading to a variety of secondary carotenoid derivatives that may no longer act as efficient antioxidants but rather as potentially harmful pro-oxidant agents. For instance, the carotenoid radical cation has strong oxidizing properties, has a relatively long lifetime (milliseconds) [31,32], and thus is able to interact with biological macromolecules [30]. 

**Figure 4:**
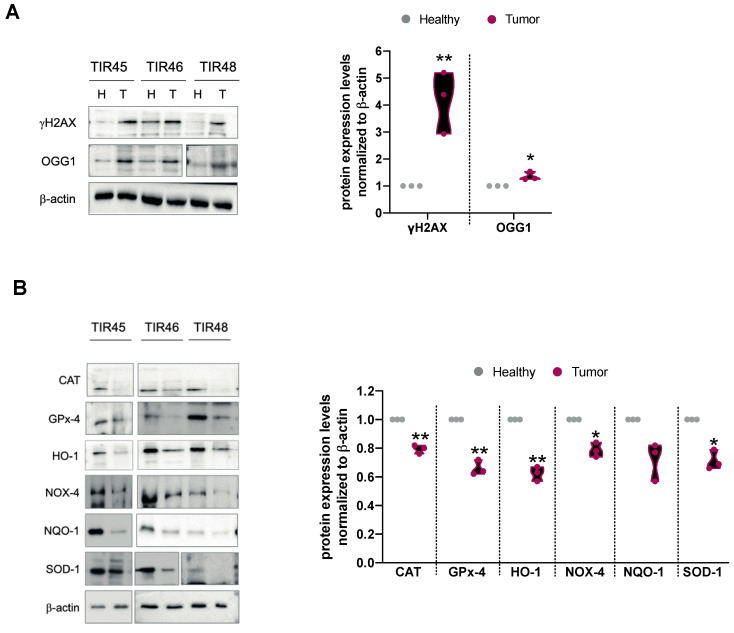
Oxidative stress and antioxidant response in PTCs. To evaluate the oxidative stress levels and the antioxidant response, protein lysates obtained from patients TIR45, TIR46, and TIR48 were analyzed by immunoblot. (**A**) Representative immunoblots are reported together with the relative quantification of pSer139-H2AX (γH2AX) and OGG1 proteins expression normalized to β-actin. Violin plots represent the mean value ±SD of the expression of each protein normalized to the healthy counterparts, in the three analyzed patients (Student’s *t*-test, * *p* < 0.05; ** *p* < 0.01 with respect to healthy lobes). (**B**) Representative immunoblots are reported together with the relative quantification of CAT, GpX-4, HO-1, NOX-4, NQO-1, and SOD-1 proteins expression normalized to β-actin. Violin plots represent the mean value ± SD of expression of each protein normalized to the healthy counterparts, in the three analyzed patients (Student’s *t*-test, * *p* < 0.05; ** *p* < 0.01 with respect to healthy lobes).

To assess whether the possible fate of the carotenoids accumulated in the PTC is associated with the oxidative stress response, we analyzed the expression of oxidative stress markers and of enzymes involved in ROS scavenging in the thyroids of three patients (i.e., TIR45, TIR46, and TIR48). The immunoblot analysis of the H2AX histone phosphorylated at Ser19 (the phosphorylated form being named γH2AX) and of oxoguanine glycosylase 1 (OGG1) showed a significant increase of both these well-known markers of genomic oxidative stress in the tumors compared to the healthy counterparts of the three analyzed PTC patients (γH2AX: 4.2-mean fold increase; t = 10.321, df = 2, *p* < 0.01; OGG1: 1.3-mean fold increase; t = 5.564, df = 2, *p* < 0.05) (Figure 4A). The Supplementary Figure S5 reports the results obtained in each analyzed patient. 

Next, we analyzed in the same patients the expression of the antioxidant enzymes. The obtained data indicate a significant reduction in the analyzed proteins in the tumor lobe compared to the relative healthy counterparts. In particular: (i) catalase (CAT) (0.2-mean fold decrease; t = 11.72, df = 2, *p* < 0.01); (ii) glutathione peroxidase-4 (Gpx-4) (0.4-mean fold decrease; t = 11.13, df = 2, *p* < 0.01); (iii) heme oxygenase-1 (HO-1) (0.4 mean fold-reduction decrease; t = 12.96, df = 2, *p* < 0.01); (iv) NADPH oxidase 4 (NOX-4) (0.2-mean fold decrease; t = 7.341, df = 2, *p* < 0.05); (v) NAD(*p*)H quinone dehydrogenase-1 (NQO-1) (0.3-mean fold decrease; t = 3.666, df = 2, not significant); (v) superoxide dismutase-1 (SOD-1) (0.3-mean fold decrease; t = 7.294, df = 2, *p* < 0.05) (Figure 4B). The Supplementary Figure S6 reports the results obtained in each patient analyzed. 

Overall, these data indicate an increased oxidative stress status and a reduced antioxidant defense response in PTCs compared to healthy thyroids.

### 3.5. Dissecting the Metabolic Status of PTCs 

**Figure 5:**
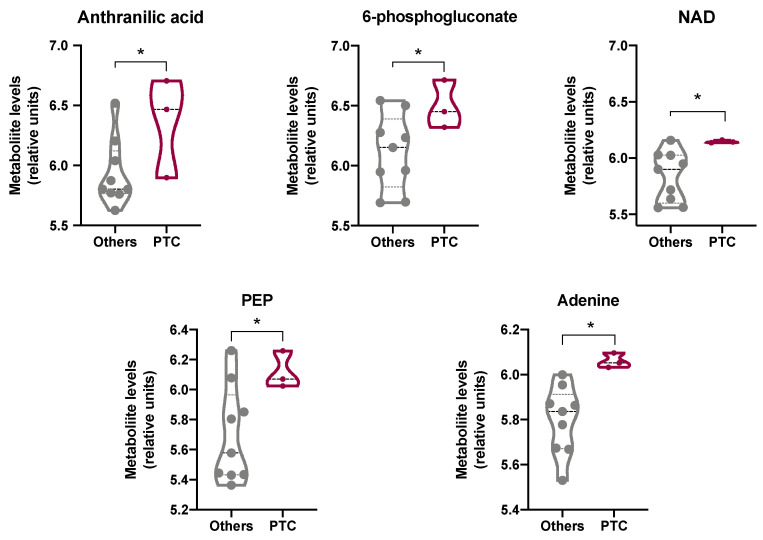
Dissection of the metabolic status of PTCs. We extrapolated from the DepMap portal (https://depmap.org/portal/; accessed on 31 January 2022) the profile of 225 metabolites in 12 thyroid cancer cell lines (i.e., three PTC (i.e., SW579, BCPAP, and BHT101), four FTC (i.e., CGTHW1, FTC238, ML1, TT2609C02), four anaplastic (i.e., 8305C, 8505C, CAL62; FTC133), one medullary (i.e., TT), and one thyroid sarcoma (i.e., S117)). Some of the metabolites involved in tryptophan metabolism (i.e., anthranilic acid, NAD, 6-phosphogluconate, adenine, and PEP) were found significantly higher in PTC cells compared to all the other thyroid cancer cell lines. Each dot in the plots represents the measured value of the indicated metabolite in one single thyroid cancer cell line (Student’s *t*-test, * *p* < 0.05 with respect to healthy lobes). Crude and analyzed data are given in Supplementary Tables S7 and S8.

With the aim of enriching the molecular characterization of PTCs and to evaluate whether PTC metabolism may be somehow differentiated from that of other TC subtypes considering the reported carotenoid accumulation [4,5], we inspected the DepMap datasets (https://depmap.org/portal/; accessed on 31 January 2022). This resource enables unbiased association analysis linking the cancer metabolome to genetic alterations, epigenetic features, and gene dependencies. In detail, we extrapolated from the DepMap portal the profile of 225 metabolites in 12 thyroid cancer cell lines (Figure 5). Three PTC (i.e., SW579, BCPAP, and BHT101), four FTC (i.e., CGTHW1, FTC238, ML1, TT2609C02), four anaplastic (i.e., 8305C, 8505C, CAL62; FTC133), one medullary (i.e., TT), and one thyroid sarcoma (i.e., S117) cell lines were profiled in the database. The data revealed that some of the metabolites involved in tryptophan metabolism were significantly higher in PTC cells compared to all the other thyroid cancer cell lines. In particular, the metabolites significantly modulated were: (i) anthranilic acid (3.8-fold increase; *p* < 0.05), a precursor to the amino acid tryptophan via the attachment of phosphoribosyl pyrophosphate to the amine group; (ii) NAD (3.2-fold induction; *p* < 0.05), an intermediate in the tryptophan-nicotinamide pathway [33]; (iii) 6-phosphogluconate (2.6-fold induction; *p* < 0.05), an intermediate in the pentose phosphate pathway that serves as an intermediate (e.g., erythrose 4-phosphate) and a cofactor in tryptophan biosynthesis [34]; and (iv) adenine (3.8-fold increase; *p* < 0.05), a nitrogen-containing base (Figure 5). Interestingly, the increased levels of anthranilic acid and NAD support a metabolism addressed toward glycolysis, as further reinforced by the statistically significant increase in phosphoenolpiruvate (PEP) observed in PTC cell lines compared to all the other thyroid cancer cells lines (2.4-fold increase; *p* < 0.05) (Figure 5). Notably, these results appear of interest because it has been reported that a high carotenoid content correlates with an increased biosynthesis of phenylalanine, tyrosine, and tryptophan ([35] (Supplementary Tables S7 and S8)). Therefore, it can be speculated that the reported accumulation of carotenoids in PTCs might possibly be linked with the increased tryptophan metabolism, which is increasingly being recognized as an important microenvironmental factor that suppresses antitumor immune response [36]. 

## 4. Discussion

Carcinogenesis is a multi-step process that results from an accumulation of injuries at several biological levels, which can include genetic and biochemical changes within cells. Our group has demonstrated that both PTC and FV-PTC are characterized by higher carotenoid levels compared to their healthy counterparts, as clearly detectable by RS [4,5]. The significant correlation between the subtype of TC and the carotenoids presence makes it possible to distinguish with the 90% of accuracy healthy tissues from and cancerous ones, as well as to discriminate between the three main high-incidence thyroid cancer variants (i.e., PTC, FTC, and FV-PTC) [4,5,6]. 

As Raman analysis does not allow us to distinguish which specific type of carotenoid accumulates in TC, here we performed HPLC analysis to dissect the profile of the carotenoids recorded in patients diagnosed with PTC. Surprisingly, the obtained results have shown that the carotenoids levels were comparable between the matched healthy and PTC lobes of all the analyzed patients. Although these findings are in contrast with the data obtained by RS, this difference can be reconciled considering the different experimental procedures of the two methods. Indeed, on one hand, RS is conducted on frozen tissue sections and allows the identification of free intracellular carotenoids. Due to their hydrophobic nature, the carotenoids released from the food matrix are dispersed into the gastrointestinal tract with the support of dietary lipids, bile-derived phospholipids, and bile salts. Thereafter, carotenoids are solubilized in the mixed micelles consisting of phospholipids, free fatty acids, monoacylglycerols, and bile salts. These solubilization steps represent a critical factor in carotenoids bioavailability and for the subsequent uptake by the intestinal cells [37]. Only once the carotenoids have been taken up by the cells does the RS analysis allow their detection in a carrier-free form into tissue sections. On the other hand, HPLC analysis requires a sample preparation in which the whole thyroid is lysed, and the proteins bound to carotenoids are degraded. Consequently, both the carotenoids localized into the vessels and those localized intracellularly can be analyzed by HPLC [38]. This implies that HPLC analysis allowed us to analyze a larger fraction of carotenoids compared to RS, indicating that their overall content in the extracellular and intracellular compartments does not differ, as expected, between the healthy and cancer thyroid lobes the same patients. On the contrary, the fraction of intracellular free carotenoids, as detected by RS, increases in PTC and FC-PTC patients [4,5,6].

As one of the major carotenoid normally found in human plasma and tissues is β-carotene [9,39], as also confirmed by the HPLC analyses of PTC patients performed here, we tested the possibility that the difference in carotenoids levels between the healthy and the PTC lobes could be due to a different metabolism of provitamin A [40]. The homeostasis of RA, which is the vitamin A derivative, is controlled by a complex metabolic pathway consisting of multiple reactions and enzymes. Briefly, dietary carotenoids such as β-carotene can be either converted to retinaldehyde (RAL) by β-carotene-15,15′-monooxygenase-1 within the enterocyte or absorbed unmodified by cells. Then, RAL can be either converted to RA by retinaldehyde dehydrogenases (RALDHs) or to retinol (ROL, also named vitamin A) by renital reductases [13,41]. Remarkably, all the above-mentioned reactions are reversible. As many tissues and organs, including the thyroid [42], express enzymes involved in the RA pathway, the β-carotene molecules delivered to these tissues can be converted in situ to retinoids [13]. Thyroid glandular cells specifically express the enzyme xanthine dehydrogenase (XDH) that, through a cellular retinoid-binding protein (CRBP)-dependent mechanism, directly oxidizes ROL to RA [42,43]. Importantly, some evidence supports a link between the thyroid gland and retinoids: both vitamin A deficiency and excess affect thyroid gland volume by affecting thyroid hormone synthesis in vivo [42,44,45]. Taking into consideration these complex pathways, if β-carotene is metabolized to RA its levels should be lower than if this metabolic pathway is inactive. Interestingly, the transcriptomic analysis performed here revealed that in all the analyzed PTC patients the overall pathways related to RA metabolism are significantly downregulated in PTC lobes compared to their healthy counterparts. This result suggests that the higher levels of carotenoids, and specifically that of β-carotene, found by RS and HPLC analyses in PTC patients, are not due to the increased uptake of carotenoids but rather to a different intracellular metabolic fate. This hypothesis is also sustained by the lack of expression of the SR-B1 receptor in PTC patients.

The beneficial and deleterious roles of the dietary carotenoids on human health may be due to several factors, but antioxidant and pro-oxidant processes seem to be particularly important. Under normal cellular conditions, carotenoids are powerful antioxidants [46,47] that act as efficient physical and chemical quenchers of singlet oxygen and as potent scavengers of other ROS [9,10,27,28,29,30]. However, the high levels of ROS that accumulate in cancer cells determine the possibility that carotenoids may act as prooxidants, causing ROS-mediated apoptosis. Indeed, the carotenoids radical products formed during the antioxidant processes can undergo further transformations, leading to a variety of secondary carotenoid derivatives that may no longer act as efficient antioxidants but rather as harmful pro-oxidant agents [30,31,48,49]. Accordingly, the well-known protective function of β-carotene and lycopene has been challenged by clinical trials where β-carotene supplementation in male smokers resulted in a significantly increased incidence of lung cancer [50]. Animal studies showed that diet influences the carcinogenic response to β-carotene and that β-carotene, per se, is pro-carcinogenic in UV carcinogenesis [51]. Interestingly, β-carotene has been shown to exhibit either limited antioxidant protection or to behave as a pro-oxidant under oxidative stress conditions [52]. Based on these considerations, we evaluated whether carotenoid accumulation in PTC patients could affect the oxidative status and the antioxidant response. Oxidative DNA damage caused by intracellular ROS is important in the pathology of a range of human diseases, including cancer [53,54,55]. The evaluation of the DNA damage levels performed here revealed the presence of significantly higher levels of DNA double-strand breaks (marked by γH2AX) in PTCs compared to the healthy counterparts. A frequently occurring mutagenic base lesion produced by ROS is 8-oxo-2′-deoxyguanosine (8-oxo-dG), which is repaired by OGG1. We found that OGG1 levels were also markedly increased in the PTC lobes of all the patients analyzed compared to the relative healthy lobes. These results agree with the observation that oxidant levels are significantly increased in patients with TC compared to the controls [56]. This high oxidative stress status agrees with data reporting increased levels of malondialdehyde (MDA) in PTCs, one of the final products of polyunsaturated fatty acid peroxidation in cells [57]. To evaluate the antioxidant response in PTC patients, the expression of six antioxidant molecules (i.e., CAT, GPx-4, HO-1, NOX-4, NQO-1, and SOD-1) was analyzed. The results obtained showed that all these antioxidant proteins and enzymes were downregulated in the PTC lobes compared to the healthy counterparts. Overall, these results agree with the oxidative stress status [58,59,60] and the decreased expression of glutathione peroxidase [60] and catalase [58,59] observed in TC, possibly contributing to the increase in the imbalance of the oxidant/antioxidant system in PTCs [61]. Interestingly, the unsupervised hierarchical clustering analysis of the gene expression profiles of the 474 PTC cases published in the framework of the TGCA project [22] indicate that the RA-related pathways resulted in being significantly downregulated in PTCs and in being associated with a worse prognosis. As we reported an increased oxidative stress status and a reduced antioxidant defense response in PTCs compared to matched healthy thyroids, it is possible to speculate that the downregulation of RA-related pathways causes carotenoid accumulation and, in turn, prooxidant effects in an oxidant microenvironment such as that present in cancer cells [30,31,48,49]. This may explain the worse prognosis of PTC patients in which the RA-related pathways result in being significantly downregulated. 

To further dissect the metabolic status of PTCs that possibly correlates with the increased levels of carotenoids, we inspected the DepMap datasets to profile 225 metabolites in 12 thyroid cancer cell lines). With this aim, three PTC (i.e., SW579, BCPAP, and BHT101), four FTC (i.e., CGTHW1, FTC238, ML1, TT2609C02), four anaplastic (i.e., 8305C, 8505C, CAL62; FTC133), one medullary (i.e., TT), and one thyroid sarcoma (i.e., S117) cell lines were analyzed. Tryptophan catabolism in cancer is increasingly being recognized as an important microenvironmental factor that suppresses antitumor immune responses. Indeed, tryptophan catabolism creates an immunosuppressive milieu by inducing T-cell anergy and apoptosis through the accumulation of immunosuppressive tryptophan catabolites [62]. In PTCs, the increased levels of tumor-associated macrophages (TAM) correlate with lymph node metastasis [63], larger tumor size [64], and reduced survival [65]. Interestingly, the data reported here revealed that some of the metabolites involved in tryptophan metabolism (i.e., anthranilic acid, NAD, 6-phosphogluconate, and adenine) were significantly higher in PTC cells compared to all the other thyroid cancer cell lines analyzed. Of note, the high levels of anthranilic acid, NAD, and PEP in PTC cell lines compared to all the other thyroid cancer cell lines support the typical tumoral glycolytic metabolism. In addition, the high carotenoid content in PTCs correlates with the reported phenylalanine, tyrosine, and tryptophan biosynthesis [35]. 

## 5. Conclusions

Overall, the results obtained here suggest that in addition to the fact that carotenoids accumulation represents a hallmark of PTCs, the downregulation of RA-related pathways also appears implicated with PTC progression and severity, possibly representing a further diagnostic hallmark for this specific type of TC. Among the limitations of this study, the relatively low number of patients enrolled should be mentioned. Therefore, this was intended as a pilot study providing novel possible markers and possible therapeutic targets for PTC diagnosis and therapy. For the future, a larger study including a higher number of PTC patients will be necessary to further validate the molecular data reported here.

## Figures and Tables

**Table 1:** List of all the patients investigated, along with clinical information. US Classification was conducted according to [14]. FA Oxy, follicular thyroid adenoma oncocytic variant; FC, follicular thyroid carcinoma; FC Oxy, follicular thyroid carcinoma oncocytic variant; FV-PTC, follicular variant-papillary thyroid carcinoma; n.a., not available; PTC, papillary thyroid carcinoma; PTC Oxy, papillary thyroid carcinoma oncocytic variant; TI-RADS, Thyroid Imaging—Reporting and Data System.

Sample	Gender	Age at Diagnosis	US Classification (TI-RADS)	Histological Diagnosis	Raman Cluster
TIR46	F	42	5	PTC	PTC
TIR47	M	52	4	FC Oxy	FC
TIR48	F	43	4	PTC Oxy	PTC
TIR49	F	45	4	PTC	PTC
TIR50	F	30	5	PTC	PTC
TIR54	F	36	4	PTC	n.a.
TIR68	F	24	4	PTC	PTC
TIR70	F	48	4	PTC	PTC
TIR77	M	45	4	PTC	FV-PTC
TIR94	M	50	5	PTC Hobnail	PTC
TIR99	M	74	3	FA Oxy	PTC

## Data Availability

We will consider sharing de-identified, individual participant-level data that underlie the results reported in this Article on receipt of a request detailing the study hypothesis and statistical analysis plan. All requests should be sent to the corresponding author. The corresponding author and lead investigators of this study will discuss all requests and make decisions about whether data sharing is appropriate based on the scientific rigor of the proposal. All applicants will be asked to sign a data access agreement. The datasets used for the hierarchical clustering and heatmap analyses used to generate Figure 3 were obtained from the Cancer Genome Atlas (TGCA) project and downloaded from cBioPortal (https://www.cbioportal.org/datasets; accessed on 26 January 2022) [22]. All these datasets are available in Supplementary Tables S5 and S6. All the datasets used to generate Figure 5 were downloaded by the Broad Institute through the DepMap portal (https://depmap.org/portal; accessed on 31 January 2022) and are available in Supplementary Tables S7 and S8.

## Supplementary Materials

The following supporting information can be downloaded at: https://www.mdpi.com/article/10.3390/antiox11081463/s1, Figure S1: Exemplificative Raman spectrum of a PTC patient, Figure S2: “Human Retinoic Acid Pathway” PCR array and heatmaps, Figure S3: Scatter plots derived from the PCR array, Figure S4: Representative immunohistochemistry image of the SR-B1 receptor expression in one PTC patient, Figure S5: Genomic oxidative stress in the three analyzed PTC patients, Figure S6: Antioxidant response in the three analyzed PTC patients, Table S1: Identification of α-carotene, β-carotene, lutein, and lycopene in the thyroids of the four patients analyzed, Table S2: List of the genes significantly modulated in the analyzed PTC patients, Table S3: Overall modulated DEGs sorted by *p*-value using the Reactome database, Table S4: Overall downregulated DEGs sorted by *p*-value using the Reactome database, Table S5: Unsupervised hierarchical clustering analysis of our dataset of “Human Retinoid Acid Pathway”-regulated genes with matched expression data in TCGA-PTC database, Table S6: Analysis of the gene expression fold change between Cluster 1 and Cluster 2 obtained from the analysis of 474 PTC cases, Table S7: Raw data extrapolated from the DepMap portal (https://depmap.org/portal/; accessed on 31 January 2022) referred to the profile of 225 metabolites in 12 thyroid cancer cell lines, Table S8: Metabolites whose levels significantly change (*p* < 0.05) between the PTC cell lines and the other thyroid cancer cell lines.
