# Supplementary material for: Unraveling the Effects of Carotenoids Accumulation in Human Papillary Thyroid Carcinoma
Source: Antioxidants (Basel). 2022 Jul 27;11(8):1463. doi: 10.3390/antiox11081463 (PMC9405418; doi:10.3390/antiox11081463)
Supplement: Supplementary file 1 [file antioxidants-11-01463-s001.zip › antioxidants-1800570-supplementary/Supplementary File_Antioxidants/DI MASI ET AL_Antioxidants_Supporting information.pdf]

Supporting information

Unraveling the effects of carotenoids accumulation in human papillary thyroid carcinoma

Alessandra di Masi[1,*], Rosario Luigi Sessa[1], Ylenia Cerrato[1], Giovanni Pastore[2], Barbara Guantario[2], Roberto Ambra[2], Michael Di Gioacchino[1], Armida Sodo[1], Martina Verri[3], Pierfilippo Crucitti[4], Filippo Longo[4], Anda Mihaela Naciu[5], Andrea Palermo[5], Chiara Taffon[3], Filippo Acconcia[1], Fabrizio Bianchi[6], Paolo Ascenzi[1], Maria Antonietta Ricci[1], Anna Crescenzi[3]

[1] Dipartimento di Scienze, Università degli Studi Roma Tre, Rome, Italy

[2] Research Centre for Food and Nutrition, CREA (Council for Agricultural Research and Economics), Rome, Italy

[3] Pathology Unit, Campus Biomedico University Hospital, Rome, Italy

[4] Unit of Thoracic Surgery, Campus Bio-Medico University, Rome, Italy

[5] Unit of Metabolic Bone and Thyroid Disorders, Fondazione Policlinico Universitario Campus Bio-Medico, Rome, Italy

[6] Fondazione IRCCS Casa Sollievo della Sofferenza, Cancer Biomarkers Unit, 71013 San Giovanni Rotondo (FG), Italy

Running title: Carotenoids and human papillary thyroid carcinoma

Keywords: antioxidant, carotenoid, papillary thyroid carcinoma

* Corresponding Author: Alessandra di Masi, PhD. email: alessandra.dimasi@uniroma3.it

Supplementary Figure S1

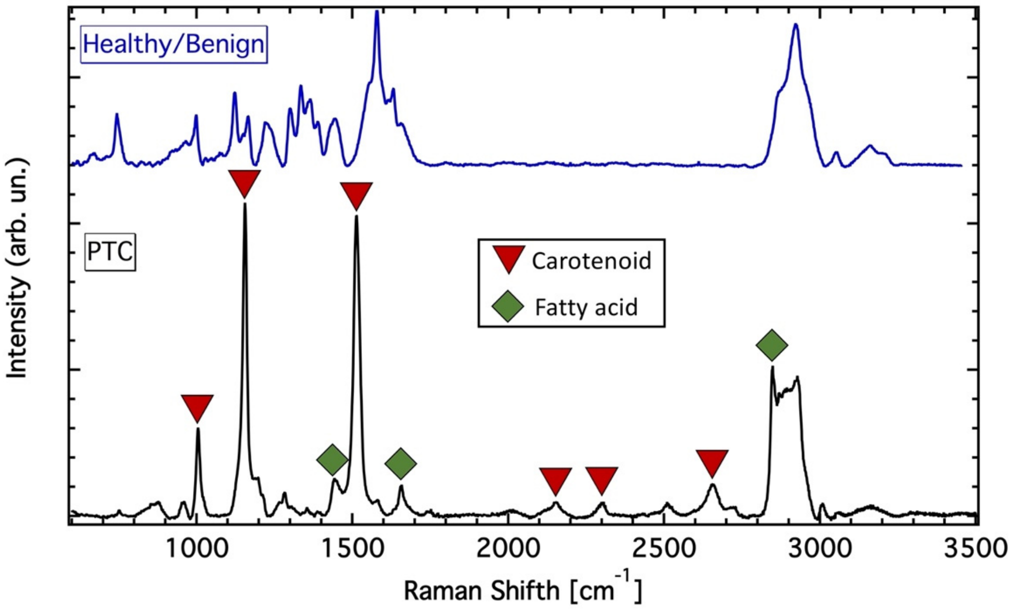

Supplementary Figure S1. Representative Raman spectrum collected from the healthy and tumor thyroid lobe sample TIR48. For more details, see [4,5].

Supplementary Figure S2

A

	1	2	3	4	5	6	7	8	9	10	11	12
A	ADH1A	ALDH1A1	ALDH1A2	ALDH1A3	APOA2	ASCL1	BHLHE40	BMP2	CD38	CDX1	CHD7	CRABP1
B	CRABP2	CYP1B1	CYP26A1	CYP26B1	CYP26C1	DCX	DHRS3	DHRS9	DLX5	EFNB1	EGR1	EPO
C	FABP5	FGF8	FOXA1	FOXG1	GATA4	GBX2	GLI1	HNF1B	HOXA1	HOXA5	HOXB1	HOXB4
D	HSD17B2	ISL1	JAG1	KLF4	LEFTY1	LHX1	LRAT	MAFB	MEIS2	MSX2	MYC	NANOG
E	NEUROD1	NRIP1	OLIG2	OTX2	PAX6	PITX2	PLAT	PPARA	PPARD	PPARG	RARA	RARB
F	RARG	RARRES3	RBP1	RBP2	RBP4	RDH10	RET	RXRA	RXRB	RXRG	SHH	SOX2
G	SOX9	SREBF1	STRA6	STRA8	TBX1	TFAP2C	TGFB2	TGM2	TUBB3	UCP1	WNT5A	WNT8A
H	ACTB	B2M	GAPDH	HPRT1	RPLP0	HGDC	RTC	RTC	RTC	PPC	PPC	PPC

B

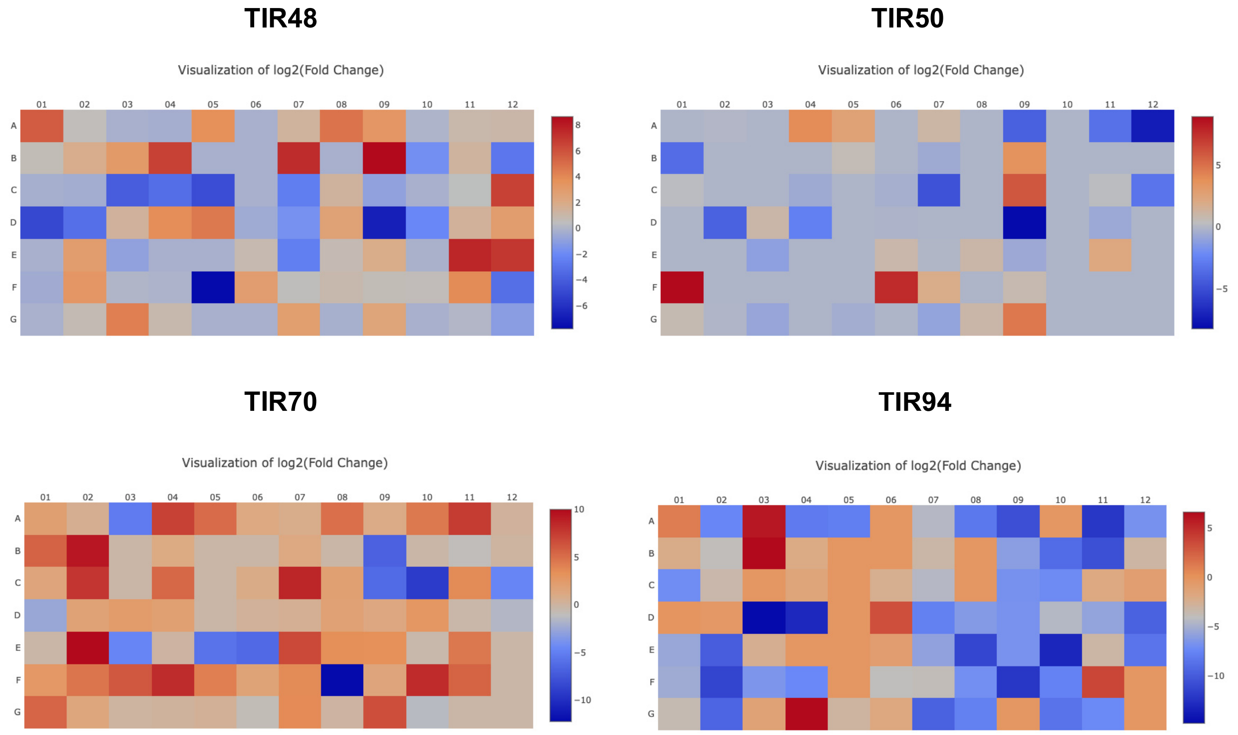

Supplementary Figure S2. "Human Retinoic Acid Pathway" PCR array (**A**) Layout of the PCR Array "Human Retinoic Acid Pathway" (Qiagen) used to evaluate the modulation of the retinoic acid signaling in matched healthy thyroid lobes and PTC counterpart. (**B**) Heatmaps obtained by comparing the expression of each gene in the PTC and healthy thyroid lobe in each of the four patients analyzed (*i.e.*, TIR48, TIR50, TIR70, and TIR94). The heatmaps were obtained using the GeneGlobe software (Qiagen).

Supplementary Figure S3

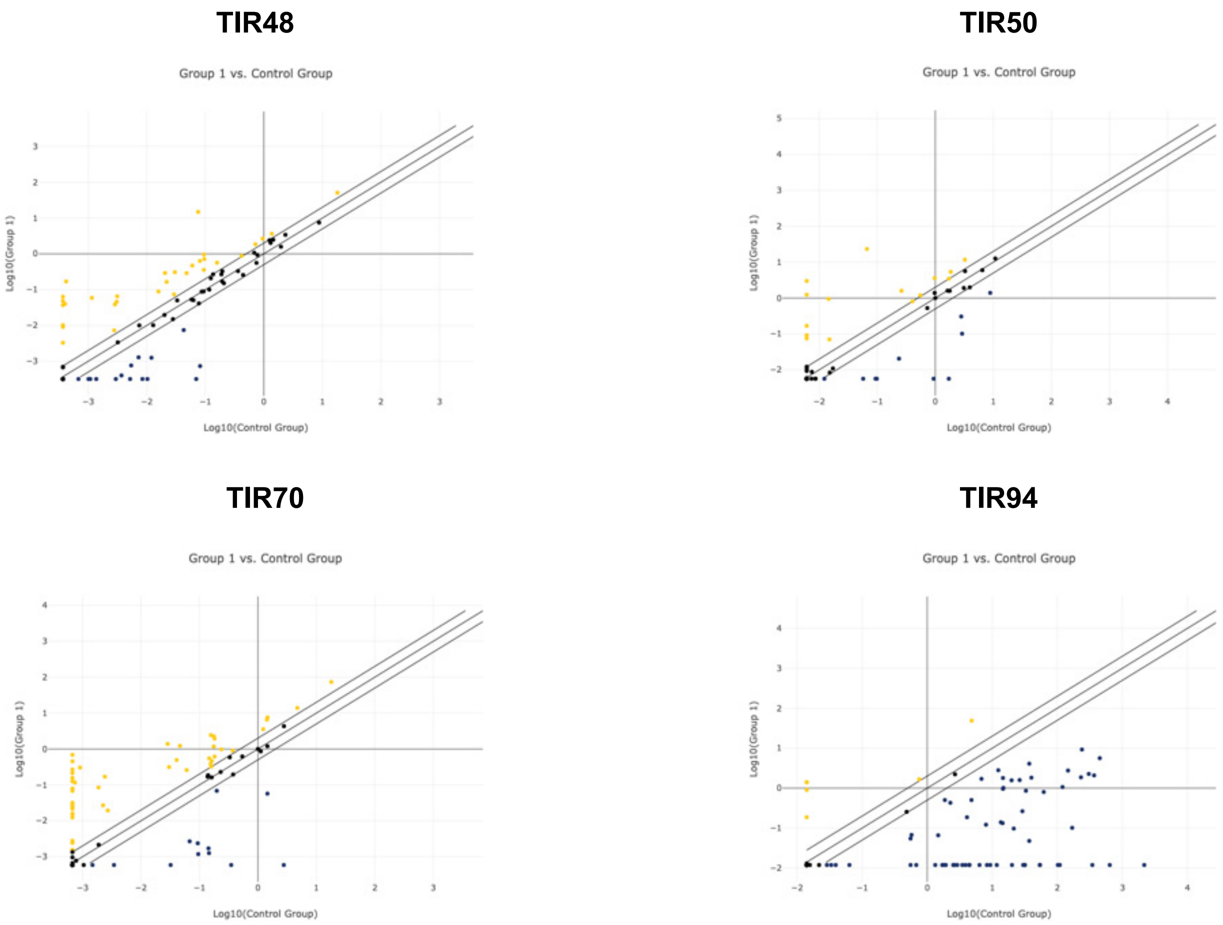

Supplementary Figure S3. **Scatter plots derived from the PCR array.** Scatter plots obtained by comparing the expression of each gene in the PTC and healthy thyroid lobe in each of the four patients analyzed (*i.e.*, TIR48, TIR50, TIR70, and TIR94). Scatter plot obtained using the GeneGlobe software (Qiagen).

Supplementary Figure S4

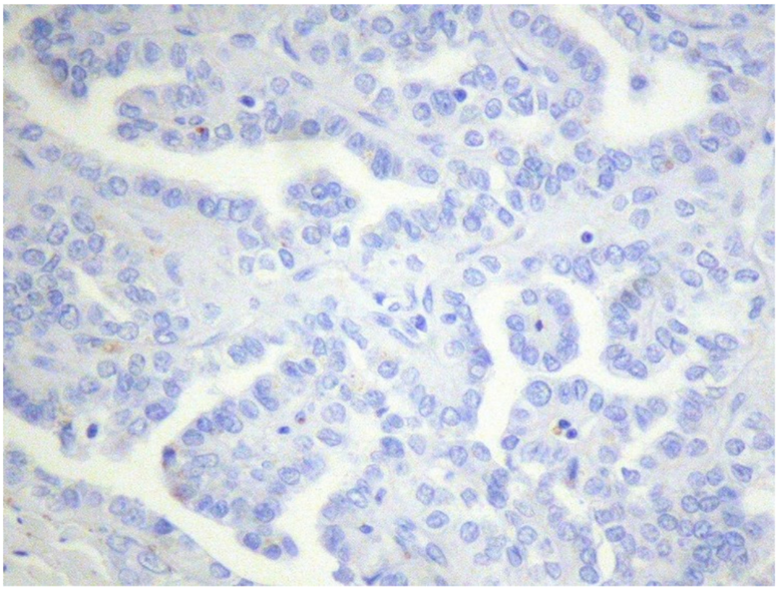

Supplementary Figure S4. **Representative immunohistochemistry image of the SR-B1 receptor expression in one PTC patients.** The image was obtained using an anti-SR-B1 polyclonal antibody. The acquisition was performed by an automatized instrument.

Supplementary Figure S5

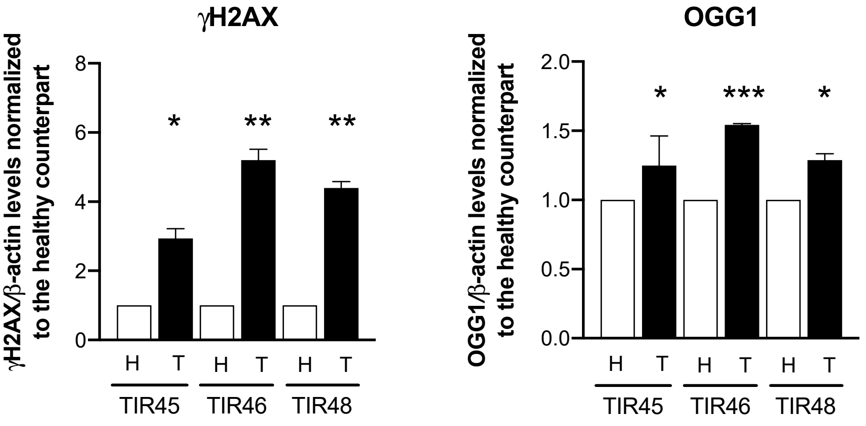

Supplementary Figure S5. Genomic oxidative stress in the three analyzed PTC patients. To evaluate the genomic oxidative stress levels, protein lysates obtained from the healthy and PTC lobes of patients TIR45, TIR46, and TIR48 were analyzed by immunoblot using the anti-γH2AX and anti-OGG-1 antibodies. The graphs represent the mean value ± SD of experiments repeated at least three times for each patient (Student's *t*-test, * $p < 0.05$; ** $p < 0.01$; *** $p < 0.001$, with respect to the relative healthy lobe). Data were normalized to the relative healthy counterpart. β-actin was used as housekeeping protein to normalize data.

Supplementary Figure S6

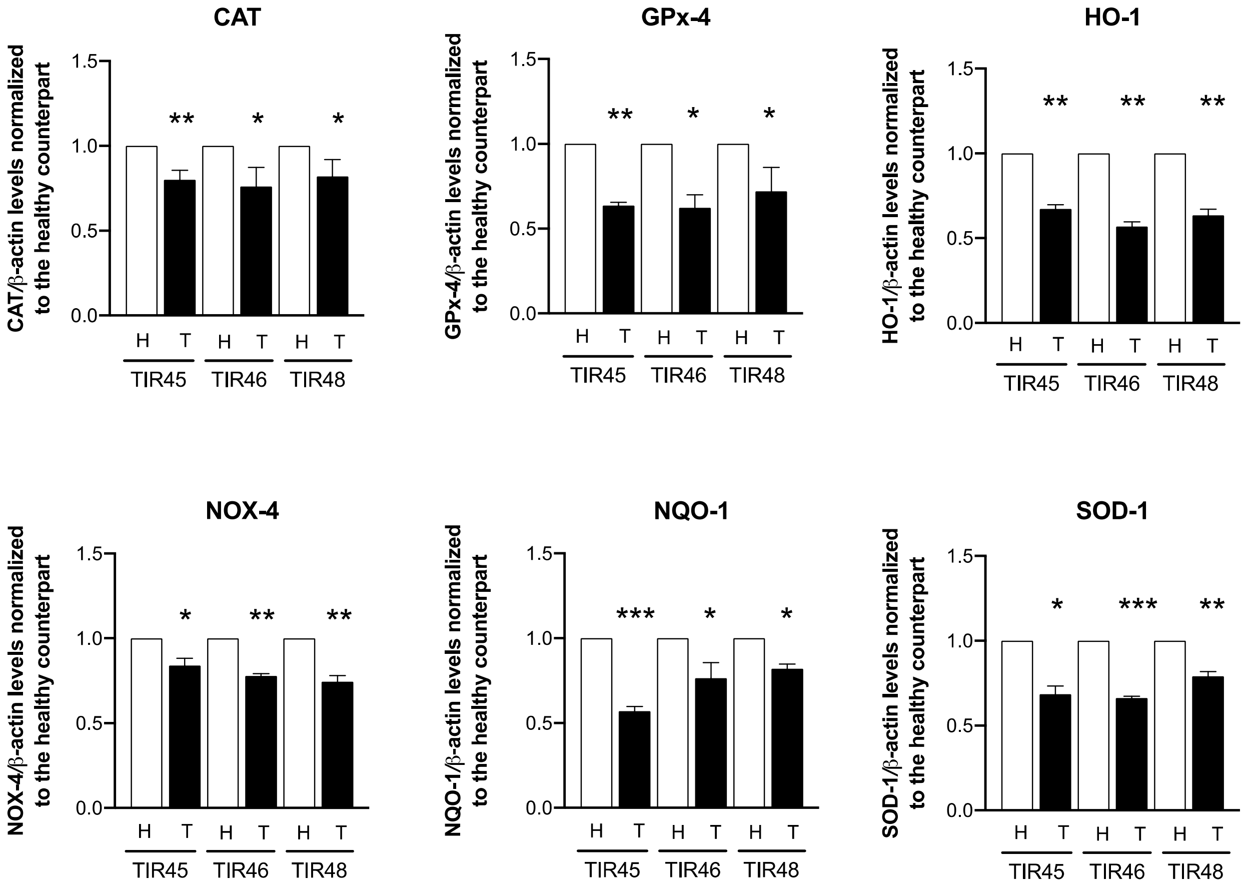

Supplementary Figure S6. Antioxidant response in the three analyzed PTC patients. To evaluate the antioxidant response, protein lysates obtained from healthy and tumor lobes of patients TIR45, TIR46, and TIR48 were analyzed by immunoblot using anti-CAT, -GpX-4, -HO-1, -NOX-4, -NQO-1, and -SOD-1 antibodies. The graphs represent the mean value ± SD of experiments repeated at least three times for each patient (Student's *t*-test, * $p < 0.05$; ** $p < 0.01$; *** $p < 0.001$, with respect to the relative healthy lobe). Data were normalized to the relative healthy counterpart reported. β-actin was used as housekeeping protein to normalize data.

Supplementary Table S1. Identification of α-carotene, β-carotene, lutein, and lycopene in the thyroids of the four patients analyzed (*i.e.*, TIR22, TIR54, TIR77, and TIR94), together with 7 unidentified peaks attributable to molecules with absorbance measured at 450 and 472 nm. Values are expressed as ng/g of thyroid tissue. H, healthy; T, tumor.

	TIR22		TIR54		TIR77		TIR99	
	**H**	**T**	**H**	**T**	**H**	**T**	**H**	**T**
**α-carotene**	16,2	9,2	52,8	46,0	53,4	39,7	23,6	22,4
**β-carotene**	57,1	92,3	222,5	213,8	141,8	150,2	136,6	131,9
**Lutein**	20,6	25,9	21,4	55,4	21,7	35,8	19,3	12,8
**Lycopene**	12,1	18,5	42,7	33,8	71,3	67,2	56,8	58,8
**Unknown 1**			25,3	32,0	67,6	35,1	0,0	0,0
**Unknown 2**			21,2	34,5	79,3	94,1	23,9	36,5
**Unknown 3**			21,2	21,7	41,0	16,5	26,1	36,7
**Unknown 4**			36,2	32,5	39,2	42,8	46,4	42,8
**Unknown 5**			45,3	42,1	44,1	41,1	30,5	32,5
**Unknown 6**			18.6	11.5	24.8	27.6	14.9	18.6
**Unknown 7**			12.7	11.8	19.7	22.7	13.2	20.1

Supplementary Table S3. Overall modulated DEGs sorted by p-value using the Reactome database. FDR, false discovery rate.

Pathway name	Entities				Reactions	
	found	ratio	p-value	FDR	found	ratio
Signaling by RA	12/73	5×10^{-3}	1×10^{-14}	4.18×10^{-12}	13/21	2×10^{-3}
Activation of HOX genes during differentiation	12/116	8×10^{-3}	2.2×10^{-12}	2.8×10^{-10}	30/43	3×10^{-3}
Activation of anterior HOX genes in hindbrain development during early embryogenesis	12/116	8×10^{-3}	2.2×10^{-12}	2.8×10^{-10}	30/43	3×10^{-3}
Signaling by nuclear receptors	18/387	2.7×10^{-2}	2.8×10^{-12}	2.8×10^{-10}	76/192	1.4×10^{-2}
Developmental biology	27/1,263	8.9×10^{-2}	2.7×10^{-10}	2.1×10^{v8}	116/556	4.1×10^{-2}
RA biosynthesis pathway	6/41	3×10^{-3}	1.2×10^{-7}	8×10^{-6}	5/11	8.1×10^{-4}
Circadian Clock	7/105	7×10^{-3}	2×10^{-6}	1.1×10^{-4}	12/59	4×10^{-3}
NR1H2 & NR1H3 regulate gene expression linked to lipogenesis	4/17	1×10^{-3}	2.7×10^{-6}	1.3×10^{-4}	8/8	5.9×10^{-4}
Generic transcription pathway	23/1,555	1.1×10^{-1}	6×10^{-6}	2.6×10^{-4}	38/830	6.1×10^{-2}
RNA Polymerase II transcription	24/1,694	1.2×10^{-1}	7.4×10^{-6}	3×10^{-4}	86/891	6.6×10^{-2}
Nuclear Receptor transcription pathway	6/86	6×10^{-3}	8.5×10^{-6}	3×10^{-4}	2/2	1.5×10^{-4}
Gene expression (Transcription)	24/1,855	1.3×10^{-2}	3.4×10^{-5}	1×10^{-3}	89/1,006	7.4×10^{-2}
PPARA activates gene expression	7/175	1.2×10^{-2}	5.2×10^{-5}	2×10^{-3}	41/41	3×10^{-3}
Regulation of lipid metabolism by PPARalpha	7/177	1.2×10^{-2}	5.5×10^{-5}	2×10^{-3}	44/44	3×10^{-3}
BMAL1:CLOCK,NPAS2 activates circadian gene expression	4/43	3×10^{-3}	9.8×10^{-5}	3×10^{-3}	4/20	1×10^{-3}
Signal transduction	30/2,997	2.1×10^{-1}	3.2×10^{-4}	8×10^{-3}	196/2,454	1.8×10^{-1}
FOXO-mediated transcription	5/110	8×10^{-3}	3.6×10^{-4}	8×10^{-3}	6/85	6×10^{-3}
TFAP2 (AP-2) family regulates transcription of other transcription factors	2/5	3.5×10^{-4}	3.6×10^{-4}	8×10^{-3}	2/2	1.5×10^{-4}
RORA activates gene expression	3/26	2×10^{-3}	4.2×10^{-4}	9×10^{-3}	3/4	3×10^{-4}
FOXO-mediated transcription of cell cycle genes	3/27	2×10^{-3}	4.6×10^{-4}	9×10^{-3}	4/22	2×10^{-3}

GLI proteins bind promoters of Hh responsive genes to promote transcription	2/8	5.6×10^{-4}	9.2×10^{-4}	1.7×10^{-3}	4/4	3×10^{-4}
NR1H2 and NR1H3-mediated signaling	4/85	6×10^{-3}	1×10^{-3}	2.3×10^{-3}	51/60	4×10^{-3}
NR1H2 & NR1H3 regulate gene expression linked to gluconeogenesis	2/10	7×10^{-4}	1×10^{-3}	2.3×10^{-3}	2/2	1.5×10^{-4}
Binding of TCF/LEF:CTNNB1 to target gene promoters	2/10	7×10^{-4}	1×10^{-3}	2.3×10^{-3}	2/4	3×10^{-4}
RUNX3 regulates WNT signaling	2/10	7.3×10^{-4}	1×10^{-3}	2.3×10^{-3}	2 5	3.7×10^{-3}

Supplementary Table S4. Overall downregulated DEGs sorted by p-value using the Reactome database. FDR, false discovery rate.

Pathway name	Entities				Reactions	
	found	ratio	p-value	FDR	found	ratio
Signaling by RA	8/73	5×10^{-3}	1.3×10^{-11}	3.3×10^{-9}	8/21	2×10^{-3}
RA biosynthesis pathway	4/41	3×10^{-3}	3.7×10^{-6}	3×10^{-4}	4/11	8.13×10^{-4}
Signaling by nuclear receptors	8/387	2.7×10^{-2}	4.5×10^{-6}	3×10^{-4}	59/192	1.4×10^{-2}
BMAL1:CLOCK,NPAS2 activates circadian gene expression	4/43	3×10^{-3}	4.5×10^{-6}	3×10^{-4}	4/20	1×10^{-3}
Developmental biology	13/1,263	8.9×10^{-2}	6.6×10^{-6}	3.5×10^{-4}	67/556	4.1×10^{-2}
Activation of anterior HOX genes in hindbrain development during early embryogenesis	5/116	8×10^{-3}	1.1×10^{-5}	4×10^{-4}	18/43	3×10^{-3}
Activation of HOX genes during differentiation	5/116	8×10^{-3}	1.1×10^{-5}	4×10^{-4}	18/43	3×10^{-3}
Nuclear receptor transcription pathway	4/86	6×10^{-3}	6.6×10^{-5}	2×10^{-3}	2/2	1.5×10^{-4}
PPARA activates gene expression	5/ 75	1.2×10^{-2}	7.6×10^{-5}	2×10^{-3}	41/41	3×10^{-3}
TFAP2 (AP-2) family regulates transcription of other transcription factors	2/5	3.5×10^{-4}	7.7×10^{-5}	2×10^{-3}	2/2	1.5×10^{-4}
Regulation of lipid metabolism by PPARalpha	5/177	1.2×10^{-2}	8×10^{-5}	2×10^{-3}	44/44	3×10^{-3}
Circadian Clock	4/105	7×10^{-3}	1.4×10^{-4}	3×10^{-3}	6/59	4×10^{-3}
RUNX3 regulates NOTCH signaling	2/16	1×10^{-3}	7.7×10^{-4}	1.5×10^{-2}	2/7	5.2×10^{-4}
Regulation of gene expression in late stage (branching morphogenesis) pancreatic bud precursor cells	2/20	1×10^{-3}	1×10^{-3}	2.2×10^{-2}	1/4	3×10^{-4}
Transcriptional regulation of testis differentiation	2/21	1×10^{-3}	1×10^{-3}	2.2×10^{-2}	14/18	1×10^{-3}
SUMOylation of intracellular receptors	2/40	3×10^{-3}	5×10^{-3}	7.4×10^{-2}	2/25	2×10^{-3}
RNA Polymerase II transcription	10/1,694	1.2×10^{-1}	8×10^{-3}	1.1×10^{-1}	9/891	6.6×10^{-2}
Transcriptional regulation by the AP-2 (TFAP2) family of transcription	2/52	4×10^{-3}	8×10^{-3}	1.1×10^{-1}	2/44	3×10^{-3}

factors						
Visual phototransduction	3/169	1.25×10^{-2}	9×10^{-3}	1.2×10^{-1}	11/92	7×10^{-3}
HHAT G278V doesn't palmitoylate Hh-Np	1/4	2.8×10^{-4}	1×10^{-2}	1.3×10^{-1}	1/1	7.4×10^{-5}
Signal transduction	14/2,997	2.15×10^{-1}	1.1×10^{-2}	0.125	102/2,454	1.8×10^{-1}
Gluconeogenesis	2/66	5×10^{-3}	1.2×10^{-2}	0.125	1/26	2×10^{-3}
Regulation of beta-cell development	2/67	5×10^{-3}	1.2×10^{-2}	0.125	3/26	2×10^{-3}
